# The Right Tool for the Job: Detection of Soil-Transmitted Helminths in Areas Co-endemic for Other Helminths

**DOI:** 10.1371/journal.pntd.0003967

**Published:** 2015-08-04

**Authors:** Maria V. Periago, Renata C. Diniz, Simone A. Pinto, Anna Yakovleva, Rodrigo Correa-Oliveira, David J. Diemert, Jeffrey M. Bethony

**Affiliations:** 1 Laboratório de Imunologia Celular e Molecular, Centro de Pesquisa René Rachou, Fundação Oswaldo Cruz, Belo Horizonte, Minas Gerais, Brazil; 2 Research Center for the Neglected Diseases of Poverty, School of Medicine and Health Science, George Washington University, Washington, DC, United States of America; 3 Department of Microbiology, Immunology and Tropical Medicine, School of Medicine and Health Science, George Washington University, Washington, DC, United States of America; University of Queensland, AUSTRALIA

## Abstract

**Background:**

Due to the recent increased use of the McMaster (MM) fecal egg counting method for assessing benzimidazole drug efficacy for treating soil-transmitted helminth (STH) infections, the aim of the current study was to determine the operational value of including the MM method alongside the Kato-Katz (KK) fecal thick smear to increase the diagnostic sensitivity when STHs are co-endemic with trematode helminths (e.g., *Schistosoma mansoni*).

**Methods:**

A cross-sectional study was conducted in school-aged children aged 4-18 years in the northeastern region of the State of Minas Gerais (Brazil), where *Necator americanus*, *Ascaris lumbricoides*, *Trichuris trichiura*, and *S*. *mansoni* are co-endemic. One fecal sample from each participant was collected and transported to the field laboratory for analysis. Coprological diagnosis was performed on each fecal sample by three different methods: Formalin-Ether Sedimentation (FES), KK and the MM technique. The diagnostic sensitivity and negative predictive value (NPV) of each technique was calculated using the combination of all three techniques as the composite standard. In order to determine the agreement between the three techniques Fleiss´ kappa was used. Both the Cure Rate (CR) and the Fecal Egg Count Reduction (FECR) were calculated using the two quantification techniques (i.e., the MM and KK).

**Results:**

Fecal samples from 1260 children were analyzed. The KK had higher diagnostic sensitivity than the MM for the detection of both *A*. *lumbricoides* (KK 97.3%, MM 69.5%) and hookworm (KK 95.1%, MM 80.8%). The CR of a single dose of mebendazole varied significantly between the KK and MM for both *A*. *lumbricoides* (p = 0.016) and hookworm (p = 0.000), with lower rates obtained with the KK. On the other hand, the FECR was very similar between both techniques for both *A*. *lumbricoides* and hookworm.

**Conclusion:**

The MM did not add any diagnostic value over the KK in areas where both STHs and trematodes were co-endemic. The lower sensitivity of the MM would have an important impact on the administration of selective school-based treatment in this area since if only the MM were used, 36 (13.9%) children diagnosed with *A*. *lumbricoides* would have gone untreated.

## Author Summary

Diagnosis of intestinal helminths and *Schistosoma mansoni* infections is based on the detection of eggs in feces. There are many techniques available for both detection and quantification of infection. For the quantification of helminth infections, the methods traditionally used are the Kato-Katz (KK) fecal think smear in humans, and the McMaster (MM) counting method in animals. Recently, the MM has been used for assessing the efficacy of benzimidazole drugs for treating soil-transmitted helminth (STH) infections in humans. In most parts of the world, however, STHs occur simultaneously with other helminth species, and the MM does not detect other helminth eggs. Therefore, in this study we sought to determine if the use of the MM in an area of Brazil were both STHs and *S*. *mansoni* are co-endemic, added any value to the current standard of diagnosis using the KK.

## Introduction

Despite numerous technological advances in the diagnosis of soil-transmitted helminth (STH) infections, such as the development of multiplex and multi-parallel fecal DNA assays [1,2], coprological microscopy techniques remain the standard for diagnosing these infections in humans [3], including the McMaster (MM) counting method [4] and the Kato-Katz (KK) fecal thick smear [5]. This is due to their simplicity, ease-of-use in the field, and low cost. Moreover, neither method requires expensive or highly calibrated instrumentation such as a real-time PCR instrument (multiplex and multi-parallel fecal DNA assay), so both can be performed on site in resource-limited endemic areas.

The KK method has been the standard technique for the detection and quantification of STH and intestinal trematode infections globally for nearly forty years as reviewed in [6–12] and is recommended by the World Health Organization (WHO) for the detection of these infections [13]. However, as pointed out by Levecke *et al*. (2011) [14], the KK method has limitations with respect to its qualitative and quantitative diagnostic performance when used to assess STHs in areas endemic for multiple helminth species. These limitations are most apparent when hookworm (e.g., *Necator americanus*) is one of the co-endemic STHs. With the use of the malachite green-glycerol solution in the KK method, helminth eggs are visualized at different time intervals or “clearing times” after the preparation of a slide. The clearing time for hookworm on a KK slide is limited to 30 minutes after preparation, whereas it is one hour for *A*. *lumbricoides* or *S*. *mansoni*. However, after 60 minutes, the glycerol solution begins to desiccates the hyaline shell of the hookworm eggs, distorting and eventually collapsing the eggs, so that they are mistaken as something else (e.g., a vegetable spore) or are reported as entirely absent, hence affecting the specificity and sensitivity of the KK for the detection hookworm infection.

In contrast, the eggs from *A*. *lumbricoides* or *S*. *mansoni* can be visualized after hours or even days after the KK slide has been prepared [15]. Moreover, the quantification of egg excretion (or the intensity of infection) by the KK method as determined by fecal egg counts (FEC) is based on a small fixed volume of feces (41.7 mg) and not on the mass of the feces submitted [5]. Hence, the quantitative performance of the KK method is controversial as the density of the feces can vary while the intensity of the eggs excreted is expressed the same, as the number of eggs per gram (EPG) of stool [16]. This potential error in FECs obtained by the KK is considered critical in programs monitoring drug efficacy, where it is thought to “introduce variation in the results and broaden the confidence levels of the resulting statistical parameters” [14]. These properties have also impeded standardization of the KK method in large-scale studies conducted at different study sites. For these reasons, the MM was used as the diagnostic method in several recent large-scale multi-site trials [17,18] of efficacy of the anthelminthics albendazole and mebendazole in treating humans for STH infections.

**Fig 1:**
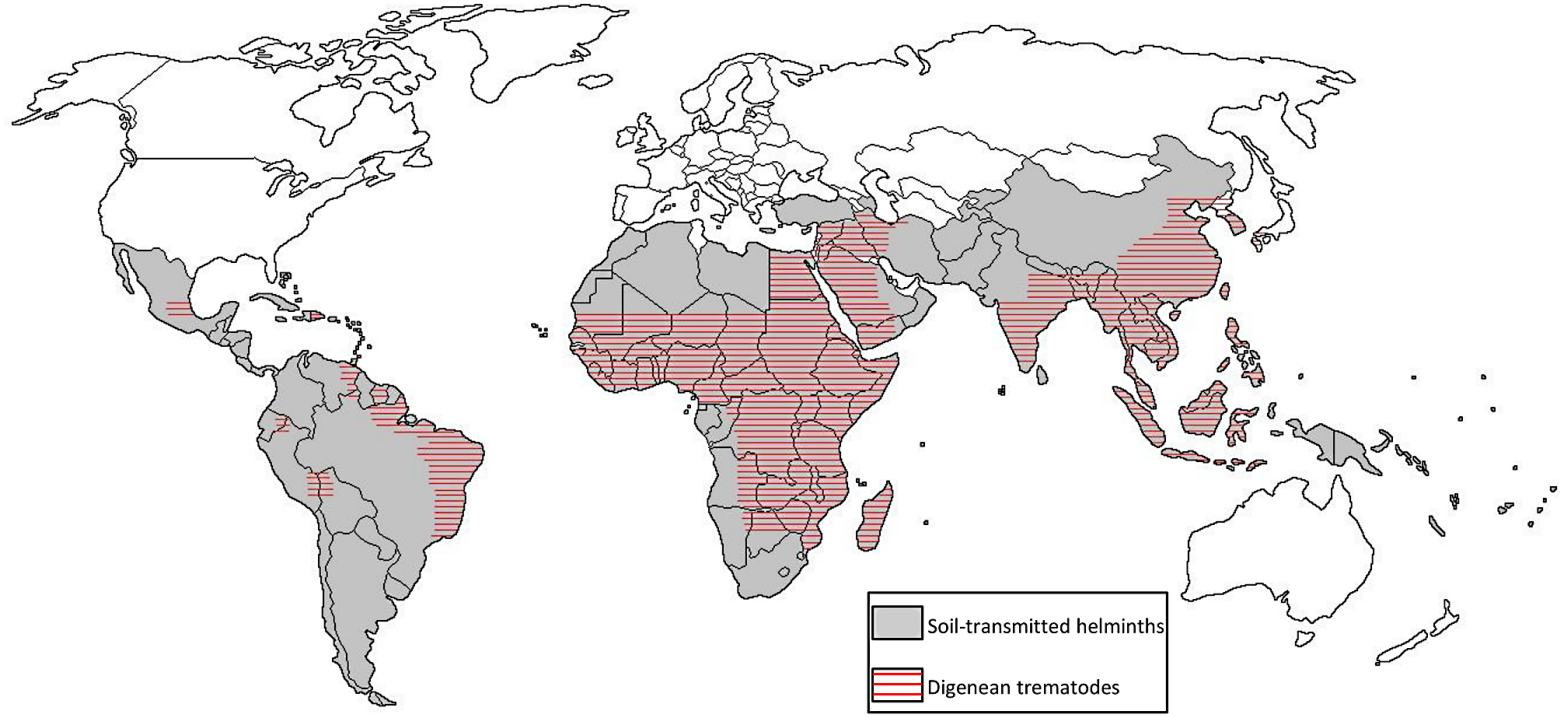
Global distribution of STHs and other trematodes species of medical importance including *Schistosoma mansoni*, *S*. *japonicum*, *Fasciola hepatica*, *F*. *gigantica*, *Fasciolopsis buski*, *Paragonimus westermani*, *Heterophyes heterophyes*, *Clonorchis sinensis* and *Opisthorchis viverrini*.

While the MM is the standard coprological method to assess STHs in veterinary parasitology, its estimates of FEC, ease of use, and suitability for poorly equipped laboratories, also make it a good choice for public health monitoring of human STHs. The aim of the current study was to determine the operational value of including the MM method in addition to the KK for routine diagnosis in areas co-endemic for several species of helminths (Fig 1). To this end, the MM and the KK methods were performed on the same fecal sample in a cross-sectional study of over 1,200 children aged 4–18 years of age in the northeastern region of the state of Minas Gerais, Brazil, where *N*. *americanus*, *A*. *lumbricoides*, *T*. *trichiura*, and *S*. *mansoni* are co-endemic, as shown in our previous studies [19,20].

More specifically, our objectives were to assess: (i) the qualitative diagnostic performance of the KK and MM (e.g., diagnostic sensitivity and negative predictive value); (ii) the quantitative diagnostic performance of the KK and MM (fecal egg counts); and (iii) the accuracy of both methods for estimating drug efficacy. While recent multinational studies have focused on the relative performances of the MM and KK for monitoring drug efficacy for treatment of STH infections [17,18], to our knowledge, this is the first study to describe the operational value of combining these two techniques for routine public health monitoring of helminth endemicity.

## Methods

### Ethics Statement

The study was approved by the National Ethics Committee of Brazil (Protocol 454/2009) and the Institutional Review Board of the Centro de Pesquisas René Rachou (Belo Horizonte, Brazil). Written informed consent was obtained from a parent or guardian of every child.

### Study Area and Study Population

This study was conducted in northeastern, Minas Gerais state, Brazil. Details of the study region have been extensively detailed elsewhere [21,22]. Prior studies have confirmed that *N*. *americanus* is the sole hookworm species endemic in this region [23,24].

### Study Design

The study was conducted as a school-based survey including children between the ages of 4 and 18 years old, inclusive. First, meetings were held with community members to explain the purpose of the study. For the parasitological survey, school personnel and students were informed 24 hours in advance of sample collection and labeled plastic containers were provided. Students were instructed to deposit one fecal sample of each child into the container and return it to school on the following day. Fresh fecal samples were transported at 4°C and those returned later than 48h after date of distribution were not accepted and new containers were issued. Refrigerated samples were transported to the nearby field laboratory located in Americaninhas, Minas Gerais state, Brazil were they were processed within 24 hours of receipt. Children positive for a STH by any of the three detection techniques described below were treated with a single oral dose of 500 mg mebendazole. Children were asked to provide a second fecal sample 7 to 14 days after treatment to monitor treatment efficacy; these samples were also analyzed by all three coprological techniques.

### Stool Examination

The presence of *A*. *lumbricoides*, *T*. *trichiura*, hookworm and *S*. *mansoni* eggs in feces was determined by trained lab personnel in compliance with standard operating procedures for the 3 different techniques routinely used in the field laboratories of the Centro de Pesquisas René Rachou: a) formalin-ether sedimentation (FES) [25]; b) KK fecal thick smear [5]; and MM counting technique [4].

#### Formalin-ether sedimentation

Fecal samples were homogenized and 3 grams were weighed and diluted in 10 ml dH2O. After mixing the feces with a wooden spatula, the contents were then filtered through a funnel containing gauze into a conical 15 ml glass tube. After the addition of 3 ml ethyl ether, the tube was capped and vigorously agitated by hand. After careful removal of the cap (done slowly to relieve any pressure build-up), the sample was centrifuged at room temperature and 1000 rpm for 1 minute. Four layers were formed with the sediment in the very bottom of the tube. The first 3 layers were decanted and then 50 μl 5% formalin was added to the sediment. Three slides were prepared from each sample by placing 50 μl of sediment between the slide and cover slip and then viewed under the microscope.

#### Kato-Katz

A solution of 3% malachite green-glycerol solution was prepared in advance and cellophane strips the size of a slide were immersed in the solution for 24 hours prior to use. After thorough homogenization of the samples, approximately 1 gram of feces was placed on a tissue paper and covered with a wire mesh. With the aid of a spatula, pressure was applied and the feces that passed through the mesh were deposited onto a standard template holding 41.7 mg of feces located on a glass slide. The template was removed and a strip of cellophane paper embedded in the 3% malachite green-glycerol solution was placed on top of the feces. Pressure was gently applied over the cellophane strip with the aid of another slide in order to spread out the sample. Two KK slides were prepared from each sample ≤24 h after receipt and examined by microscope using 100x magnification after 30–60 minutes of clearing time. Each slide was observed in its entirety and for each sample; the EPG of feces was obtained by multiplying the mean number of eggs per slide by 24. The clearing time is defined as the period after the application of the malachite green-glycerol solution until eggs can be visualized. Due to the osmotic force on the thin hyaline layer of hookworm eggs, the slide must be read within 30–60 minutes after preparation or these eggs will desiccate rapidly and become deformed beyond recognition as the KK slide dries. Nonetheless, during that time interval, it is possible to detect and quantify eggs from all three STHs and *S*. *mansoni*.

#### McMaster counting technique

Two grams of homogenized fresh fecal sample were suspended in 30 ml saturated sodium chloride (NaCl) solution (specific gravity [SG] = 1.22) prepared in advance by dissolving 333 g of NaCl in 1L of distilled water and observing precipitation. The suspension was poured three times through a wire mesh with the aid of a wooden spatula to remove large debris. The filtered portion of the sample was then homogenized for one minute using a magnetic bar and stirrer. Immediately after stirring, 0.5 ml aliquots were removed from the very top surface of the supernatant using a Pasteur pipette and then added into 2 chambers of a MM slide. The filled chambers were allowed to stand for two minutes and were examined under a light microscope using 100x magnification and the EPG of feces for each helminth species was obtained by multiplying the total number of eggs counted on both chambers by 50. Of note, the MM technique does not detect eggs of *S*. *mansoni*.

### Statistical Analysis

All data were double-entered in Microsoft Excel (2010 Microsoft Corporation). Statistical analyses were performed using SPSS Statistics for Windows, Version 19.0 (Copyright IBM Corporation 1994, 2013) and GraphPad Prism, Version 6.0 (2015 GraphPad Software, Inc.)

#### Diagnostic sensitivity and egg counts

Intensities of infection for the STHs and *S*. *mansoni* were stratified according to WHO guidelines into light, moderate or heavy categories [26]; for *A*. *lumbricoides* these were defined as between 1–4,999, 5,000–49,999, and ≥50,000 EPG, respectively; for *T*. *trichiura*, 1–999, 1,000–9,999, and ≥10,000 EPG; for *N*. *americanus*, 1–1,999, 2,000–3,999, and ≥4,000 EPG; and, for *S*. *mansoni*, 1–99, 100–399, and ≥400 EPG. A related-samples Wilcoxon Ranked Signed test was used to evaluate differences in mean EPG counts obtained by the KK and MM methods. Scatter plots were used to assess the linearity of the relationship between average eggs counts and their correlation was analyzed using Spearman’s correlation coefficient.

Following the methods used by [14,27–38], the sensitivity and negative predictive value (NPV) were assessed for each diagnostic technique in comparison to a composite reference standard, which was defined as being positive if any of the 3 tests were positive. The specificity of the composite standard was assumed to be 100% given the small number of false positives found when experienced technicians examine the slides microscopically [39]. The sensitivity of both the KK and MM were also calculated by intensity class and compared against the composite reference standard.

The degree of agreement between the different diagnostic techniques (compared two by two) was assessed by calculating Cohen’s kappa (κ) statistic [40] and the agreement between all three techniques was assessed by calculating Fleiss’ kappa statistic [41], which allows comparison between more than two raters. The following cut-offs were used to calculate the degree of agreement: κ < 0, poor agreement; κ between 0 and 0.20, slight agreement; κ between 0.21 and 0.40, fair agreement; κ between 0.41 and 0.60, moderate agreement; κ between 0.61 and 0.80, substantial agreement; and, κ between 0.81 and 1.0, almost perfect agreement. For the KK and MM, Cohen’s kappa statistic was also used to assess the agreement in the assignment of the cases to the different classes of infection intensity (e.g., light, moderate or heavy) according to WHO guidelines [26].

#### Drug efficacy

Both the Cure Rate (CR) and the Fecal Egg Count Reduction (FECR) were calculated using the two quantification techniques (i.e., the MM and KK). The CR measures the percent of those treated who become egg negative after drug administration and is calculated by dividing the number of individuals who are egg positive post-treatment by the number who are positive pre-treatment and then multiplying by 100 [42]. The calculated CRs were compared using a two-sample test of proportion, setting the significance level at p < 0.05.

$$CR\;=\;100\text{\% * }(1-\;\frac{number\;of\;subjects\;excreting\;eggs\;at\;follow-up}{number\;of\;subjects\;excreting\;eggs\;at\;baseline}$$

The FECR measures the percent reduction in egg intensity after drug treatment using the arithmetic mean (AR) of pre- and post-treatment egg counts. It is calculated by subtracting the AR of post-treatment FECs from the pre-treatment FECs, dividing it by the AR of pre-treatment FEC and then multiplying by 100 [17].

$$FECR\;\left(\text{\%}\right)\;=\;100\text{\% * (}1-\frac{ARmean\;\left(post-FEC\right)}{ARmean\;\left(pre-FEC\right)})$$

## Results

### Study Population

A total of 1,260 children from 23 schools were included in the study, of which 635 (50.4%) were female and 625 (49.6%) were male. The ages ranged from 4 to 18 years of age, with a mean age of 10.1 years. All the children included in the study provided a sufficient volume of feces to perform all 3 diagnostic procedures. A total of 311 children were found to be positive for at least 1 of the 3 STHs and were therefore treated with a single dose of 500 mg mebendazole. Of these, 302 provided fecal samples at follow-up 7–14 days following treatment.

### Prevalence of Helminth Species

A total of 312 children were infected with *S*. *mansoni*. Since the MM is a flotation technique and therefore does not detect eggs of *S*. *mansoni*, the overall prevalence for this helminth (24.8%) was calculated using only the FES and KK techniques. Moreover, 46.2% of the children diagnosed with *S*. *mansoni* were co-infected with at least 1 STH. By combining the results from the 3 different techniques into a composite standard outcome measure, the prevalence for each STH was: 22.8% for hookworm, 20.6% for *A*. *lumbricoides* and 1.2% for *T*. *trichiura*. The overall prevalence and the individual prevalence obtained using each technique is detailed in Table 1.

**Table 1:** Prevalence, sensitivity and negative predictive value (NPV) derived by the different diagnostic methods for STHs and *S*. *mansoni*.

Parasite	Technique	Prevalence (%)	Sensitivity (%)	NPV (%)
**Hookworm**	Composite*	22.8	100	100
	Formalin-Ether Sedimentation	21.8	95.8	98.8
	Kato-Katz	21.7	95.1	98.6
	McMaster	18.4	80.8	94.6
***A*. *lumbricoides***	Composite*	20.6	100	100
	Formalin-Ether Sedimentation	19.4	94.2	98.5
	Kato-Katz	20.0	97.3	99.3
	McMaster	14.3	69.5	92.7
***T*. *trichiura***	Composite*	1.2	100	100
	Formalin-Ether Sedimentation	1.0	86.7	99.8
	Kato-Katz	1.0	86.7	99.8
	McMaster	0.6	46.7	99.4
***S*. *mansoni***	Composite*	24.8	100	100
	Formalin-Ether Sedimentation	20.4	82.4	94.5
	Kato-Katz	23.7	95.5	98.5
	McMaster	-	-	-

*Composite: positive result from any of the three coprological tests.

### Method Comparison for the Diagnosis of STHs and *S*. *mansoni*

Table 1 summarizes the relative performance characteristics of the 3 different fecal techniques used for the diagnosis of STH infections. Although the technique with the best performance for the diagnosis of hookworm proved to be FES, the kappa statistic (Table 2) showed an almost perfect agreement between all 3 techniques, with a Fleiss’ κ of 0.887. On the other hand, for *A*. *lumbricoides*, the technique with the best performance was the KK. Again, Fleiss’ kappa statistics comparing the techniques showed almost perfect agreement between them with a κ of 0.843, although Cohen’s kappa statistics showed only substantial agreement between the KK and MM (κ = 0.794) and between the FES and MM (κ = 0.791). Regarding the diagnosis of *T*. *trichiura*, both FES and KK were equally useful, with almost perfect agreement between them (Cohen’s kappa of 0.845). On the other hand, the agreement between the KK and MM techniques and between the MM and FES was only substantial (κ = 0.629 and 0.698, respectively). The overall agreement between all 3 techniques for *T*. *trichiura* as measured by Fleiss’ kappa was of substantial agreement (κ = 0.694). The comparison of the 2 techniques used for the diagnosis of *S*. *mansoni* demonstrated that the KK performed better than the FES, although Cohen’s kappa statistic showed almost perfect agreement between them with a κ of 0.841 (Table 2).

**Table 2:** Agreement between the different diagnostic techniques for the detection of STHs and *S*. *mansoni*.

Parasite	Techniques compared	Cohen’s Kappa (κ)	Fleiss’ Kappa (κ)*
**Hookworm**	KK vs FES	0.944	
	KK vs MM	0.854	0.887
	MM vs FES	0.860	
***A*. *lumbricoides***	KK vs FES	0.935	
	KK vs MM	0.794	0.843
	MM vs FES	0.791	
***T*. *trichiura***	KK vs FES	0.845	
	KK vs MM	0.629	0.694
	MM vs FES	0.698	
***S*. *mansoni***	KK vs FES	0.841	

KK = Kato-Katz direct fecal smear; MM = McMaster counting technique; FES = Formalin-Ether Sedimentation.

*Used for the comparison of all three techniques.

### Quantitative Fecal Egg Counts

**Fig 2:**
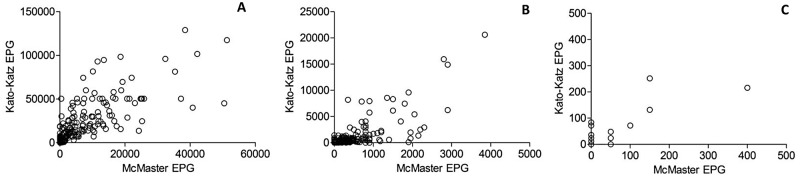
Scatter plots of arithmetic egg counts (eggs per gram of feces—EPG) comparing the Kato-Katz direct fecal smear (KK) and the McMaster counting method (MM). The graphs are presented separately for each soil-transmitted helminths: A. *Ascaris lumbricoides*, B. Hookworm, C. *Trichuris trichiura*. Correlation was measures using Spearman’s correlation coefficient: 0.86 for *A*. *lumbricoides*, 0.88 for hookworm and 0.63 for *T*. *trichiura*.

The maximum EPG count, geometric and arithmetic mean of the EPG counts, standard deviation from the mean, and lower and upper 95% confidence intervals for STHs and *S*. *mansoni* obtained with the KK and MM counting techniques are summarized in Table 3. This table also summarizes the infection intensities of the positive samples, stratified into light, moderate or heavy infections. The mean EPG was significantly higher with the KK than the MM, as evaluated using the related-samples Wilcoxon Ranked Signed Test (p<0.001), for hookworm (207.39 vs. 86.53 EPG) and *A*. *lumbricoides* (3940.01 vs. 1329.33 EPG) but not for *T*. *trichiura* (p = 0.396). The maximum EPG value was lower with the KK for *T*. *trichiura* (252 vs. 400 EPG), although it was higher for both *A*. *lumbricoides* (129,120 vs. 51,250 EPG) and for hookworm (20,616 vs. 3850 EPG). The relationship between the EPG values obtained for each sample by each technique followed a linear trend as shown by scatter plots presented in Fig 2. The correlation between the KK and MM based on Spearman’s correlation coefficient was significant for all three STHs (0.86 for *A*. *lumbricoides*, 0.88 for hookworm and 0.63 for *T*. *trichiura*).

**Table 3:** Characteristics of STH and *S*. *mansoni* infections among children in Americaninhas, Minas Gerais, Brazil, as determined by the Kato-Katz (KK) and McMaster counting method (MM).

Parasite	Arithmetic Mean EPG	Max EPG count	SD	Lower, Upper 95% CI	GeoMean EPG	Wilcoxon Ranked Signed test	Method
Hookworm	207.4	20616	1149	143, 270.	285.0	<0.001	KK
	86.5	3850	304	69, 103	282.9		MM
*A*. *lumbricoides*	3940.0	129120	13254	3940, 3207	6564.5	<0.001	KK
	1329.3	51250	5065	1049, 1609	4016.5		MM
*T*. *trichiura*	0.83	252	11	0.22–1.44	55.6	0.396	KK
	0.75	400	13	0.02–1.49	101.7		MM
*S*. *mansoni*	76.5	10272	481	50–103	84.2	NA	KK

EPG refers to eggs per gram of feces. SD refers to the standard deviation of the arithmetic mean. CI refers to Confidence interval. Geomean refers to geometric mean.

Moreover, when the sensitivity of the KK and MM was calculated according to the classification by infection intensity, the KK proved to be more sensitive than the MM for light, medium and heavy infections. This was true for all three STHs detected in the study (Table 4).

**Table 4:** Diagnostic sensitivity of the Kato-Katz (KK) and McMaster (MM) techniques according to intensity of infection.

Intensity of infection			Parasite			
	*A*. *lumbricoides*		Hookworm		T. trichiura	
Method	MM	KK	MM	KK	MM	KK
Light	51.2	88.9	79.1	93.3	42.9	78.6
Moderate	52.9	92.3	12.1	43.3	-	-
Heavy	2.4	79.6	-	48.5	-	-

In general, the KK technique detected more cases of moderate and heavy intensity infections when compared to the MM counting technique (Table 5). Using Cohen’s kappa statistics (κ), the agreement in the classification between the MM and KK for the STHs showed differences depending on the species being studied (Table 5). For hookworm, the agreement was substantial (κ = 0.70) for light-intensity infections, slight (κ = 0.11) for moderate-intensity infections and poor (κ = 0.00) for heavy-intensity infections. For *Ascaris*, the agreement for both light- and heavy-intensity infections was slight (κ = 0.20 and 0.02, respectively) while for moderate-intensity infections it was fair (κ = 0.33). The difference observed in *A*. *lumbricoides* infection intensities is probably due to the presence of infertile *A*. *lumbricoides* eggs since the MM does not detect them because they are too heavy to float. During this study, the presence of infertile *A*. *lumbricoides* eggs was annotated and it was observed that the MM does not detect them. In 41 of the 259 cases of *A*. *lumbricoides* that were detected (15.8% of all cases detected); the presence of infertile eggs was observed, but only by the KK method. In 5 of these samples, the MM detected only fertile eggs while the KK detected both fertile and unfertile eggs; the remaining 36 cases that contained only infertile eggs were missed by the MM. For *Trichuris*, only light-intensity infections were diagnosed by both methods and the agreement between them was slight (κ = 0.19).

**Table 5:** Cohen’s kappa statistics (κ) to evaluate the agreement in the classification of STHs according to classes of intensity as determined by the Kato-Katz direct fecal smear (KK) and McMaster counting method (MM).

	No. of children stratified by infection intensity				
Parasite	Negative	Light	Moderate	Heavy	Method
**Hookworm**	987	244	13	16	KK
	1028	224	8	0	MM
**Proportions of Agreement (κ)**	**0.94**	**0.70**	**0.11**	**0.00**	
***A*. *lumbricoides***	1008	94	121	37	KK
	1080	88	90	2	MM
**Proportions of Agreement (κ)**	**0.93**	**0.20**	**0.33**	**0.02**	
***T*. *trichiura***	1247	13	0	0	KK
	1253	7	0	0	MM
**Proportions of Agreement (κ)**	**0.99**	**0.43**	**NA**	**NA**	

NA: Not applicable

### Efficacy of Treatment

As shown in Table 6, the CR was significantly lower when assessed by the KK technique in comparison to the MM for both hookworm (p < 0.001) and *A*. *lumbricoides* (p = 0.016). Therefore, 22 of the 189 children (11.6%) who were treated were determined to be cured from *A*. *lumbricoides* by the MM technique but were in fact still infected as determined by the KK. Also, after treatment, the MM failed to detect 38 light hookworm infections out of the 212 children treated (17.9%) that were diagnosed using the KK. On the other hand, 4 children (1.9%) with light hookworm infections after treatment went undetected by the KK method but were detected by the MM. Nonetheless, this difference in egg detection did not have an impact on the FECR since it was very similar between both techniques. The CR and FECR for *T*. *trichiura* were not calculated due to the small number of positive samples found.

**Table 6:** Cure Rates (CR) and Fecal Egg Count Reductions (FECR) after treatment with Mebendazole.

	McMaster Counting Method		Kato-Katz Direct Fecal Smear	
STH	CR (%)	FECR (%)	CR (%)	FECR (%)
Hookworm	61.0^a^	84.0	32.0^a^	86.0
*A*. *lumbricoides*	74.0^b^	97.0	59.0^b^	96.0

^a^ Two sample test of proportions: p < 0.001

^b^ Two sample test of proportions: p = 0.016

## Discussion

The McMaster counting method (MM) is an inexpensive and easily implemented flotation technique, extensively used in veterinary parasitology and, more recently, in human studies to estimate cure rates for anthelminthics of the benzimidazole class, including albendazole [18] and mebendazole [17]. While the diagnostic sensitivity and specificity of the MM method for detecting nematode infections has long been reported to be excellent in humans and animals, its recent application in multi-nation studies for monitoring drug efficacy for STHs prompted us to consider incorporating it as a routine component in our epidemiological and public health surveys for STH infections in endemic areas of northeastern Minas Gerais state, Brazil [21–24,43]. As many regions are co-endemic for STHs and the trematode *S*. *mansoni*, which cannot be detected by the conventional MM, the principal rationale for adding the MM into our survey workflow for STH infections would be improved diagnostic sensitivity. Hence, the objective of the current study was to assess the added value of a combination detection strategy, which includes both the MM and KK methods.

Overall, the observed diagnostic performances of the MM and KK in this study were comparable for STHs, which is consistent with prior studies including a recent meta-analysis [44], and alone argues against the extra cost, time, and labor of including the MM in our diagnostic workflow. However, of particular concern was our observation that the MM method fell far short of its intended use in our combined diagnostic strategy in some critical respects, most importantly leading to several false negative results. In fact, the KK outperformed the MM for each of the three STHs studied, especially for the detection of *A*. *lumbricoides* eggs, for which there was a marked difference in egg detection between the 2 methods (Table 1). The KK method was far more sensitive (97.3%) for diagnosing *A*. *lumbricoides* infections than the MM method (69.5%), with the KK method detecting 36 more *A*. *lumbricoides* cases than the MM method. Although this difference in sensitivity has already been observed in other comparative studies [14,31,32,45], this is the first time that the extent that infertile *A*. *lumbricoides* eggs are undetected by the MM has been documented. A marked difference between the two methods was also observed when classifying *A*. *lumbricoides* infections by intensity according to WHO guidelines [13] since the KK method classified more cases into the moderate (κ = 0.33, fair agreement) and heavy (κ = 0.02, slight agreement) categories.

The reason for the difference in the performance of the two methods in regards to *A*. *lumbricoides* lay mainly with the presence of infertile *Ascaris* eggs in human feces. This is due to the fundamental mechanism of the conventional saline used in the MM, which relies on suspending a homogenized fecal sample in a NaCl solution that is pipetted into a chambered slide, where the specific gravity of the solution “floats” STH eggs to the chamber’s upper surface, where they can be counted under low magnification (usually 100X). While the MM method readily detects fertilized *A*. *lumbricoides* eggs given their specific gravity of 1.13 [46] (which is well within range of flotation for the MM method), it does not detect infertile *A*. *lumbricoides* eggs. The latter observation is due to a combination of both the high specific gravity (SG = 1.18) of infertile *A*. *lumbricoides* eggs [47], which is outside the range of flotation for the MM method, and the absence of a lipoidal membrane that prevents the equilibration of the interior of the egg with the external flotation media [47], so that infertile eggs sink instead of float. This deficiency in the MM method had a significant impact on the diagnosis of this STH in the current study: if the MM method had been used without the KK, the presence of *A*. *lumbricoides* eggs would have gone undetected in 36 of 259 children with this infection. Moreover, as shown in Table 6, the CR for *A*. *lumbricoides* was significantly lower when assessed using the KK method given that the MM method failed to detect the presence of eggs in the fecal samples of 22 children that had been treated with mebendazole.

The use of the MM method for the detection of hookworm eggs also added little extra diagnostic value to our study since the KK was found to be more sensitive (95.1%) than the MM (80.8%), although the best performance characteristics were obtained with FES (95.8%) (Table 1). For the quantification of hookworm infection, the KK method was able to detect not only more light intensity hookworm infections than the MM method, but also more moderate and heavy infections. This difference was statistically significant as shown by the kappa statistic (Table 5) since the agreement between the techniques for moderate and heavy infections was “slight” and “poor”, respectively. Moreover, similar to our observations for *A*. *lumbricoides*, the CR for hookworm was significantly lower when the KK method was used since the MM method failed to detect the presence of eggs in the fecal samples of 38 children (17.9%) that had been treated with mebendazole. It must be pointed out, however, that the KK failed to detect hookworm eggs that were observed by MM in 4 children who had been treated.

Finally, the strongest agreement between the 3 helminth detection techniques studied was found between the FES and KK techniques. The advantage of using both the FES and KK techniques is that it allows the detection of both protozoa and helminths (using the non-quantitative FES) and the quantification of several helminth species (using the KK), including *S*. *mansoni*, an important helminth in our area of study (S1 Fig). Based on our experience and the results reported herein, we recommend the use of both of these techniques in areas where STHs and *S*. *mansoni* are endemic, with the FES used as a screening test and KK used to quantifying infections. Furthermore, we do not recommend relying solely on the single solution MM for the study of STHs due to the important implications on treatment. In our opinion, the use of the KK would be a better choice for STHs and a more useful one from a public health perspective, minimizing the number of false negatives, ensuring treatment of infected individuals and, consequently, minimizing transmission.

Our analysis is subject to several limitations. First, our evaluation of helminths diagnostic test performance was done in the absence of a “gold” standard to estimate the ‘true’ unmeasured infection status and allowing for conditional dependency between the test outcomes. Numerous evaluations of STH diagnostics have relied on a composite reference standard as done in the current study, which is generated by combining the results of the MM, KK and the FEC. However, this approach has been widely criticized [48,49], with the absence of an accepted gold standard being a major obstacle for comparative evaluations of such diagnostic tests. In a recent meta-analysis [44], the authors addressed this problem by using Bayesian latent class analysis, enabling simultaneous estimation of the unknown true prevalence of infection and the sensitivities and specificities of compared diagnostic tests, which was a computational method beyond our means. Another limitation is that we could not evaluate the operational value of using the MM method for the detection of *T*. *trichiura* because the prevalence of this STH was low (1.5%) in the study sample. Finally, a fairer comparison between the MM and the KK methods would have been to use a zinc sulphate (ZnSO4(H2O)7 + H2) flotation solution in the MM, which would have enabled the detection of trematode eggs in an MM counting chamber, much like a mini-FLOTAC, which has been positively evaluated for both protozoa and helminth infections in humans, including *S*. *mansoni* [38,50,51]. However, the mini- FLOTAC was unavailable to us as the time of our study, and we look forward to a greater availability after it has gone through further validation studies before we can incorporate it into our public health surveys.

### Conclusion

Based on the diagnostic sensitivities of the MM and the KK as reported herein, we do not see the value of the extra time, materials, and labor required by the addition of the MM into a diagnostic strategy for STHs in areas where *S*. *mansoni* infection is also present, especially resource limited areas such as our study site in Brazil. Initially, we hypothesized that the MM method would improve the sensitivity for the detection of STHs over the use of the KK alone, especially for the diagnosis of hookworm infections. However, as shown here, the inclusion of the MM in our diagnostic workflow did not improve overall sensitivity. Similarly, the MM could not replace the KK in the study area given its lower sensitivity for diagnosing *A*. *lumbricoides* and its inability to detect *S*. *mansoni* eggs. Nonetheless, when conducting coprological studies in humans, determining which technique to use will depend on the research question, the expected species of interest, the expected prevalence and intensity of each species and finally, the field conditions for implementation—in other words, “finding the right tool for the job”.

## Supporting Information

S1 Fig Gold Standard for co-endemic areas using the Formalin-Ether Sedimentation for detection of helminths and the Kato-Katz direct fecal smear for their quantification. This standard could be used regardless of co-endemnicity.
(TIF)

S1 Checklist STARD checklist. (DOC)

S1 Flowchart STARD flowchart. (DOC)
