# Supplementary figures and images for: The Right Tool for the Job: Detection of Soil-Transmitted Helminths in Areas Co-endemic for Other Helminths
Source: PLoS Negl Trop Dis. 2015 Aug 4;9(8):e0003967. doi: 10.1371/journal.pntd.0003967 (PMC4524677; doi:10.1371/journal.pntd.0003967)

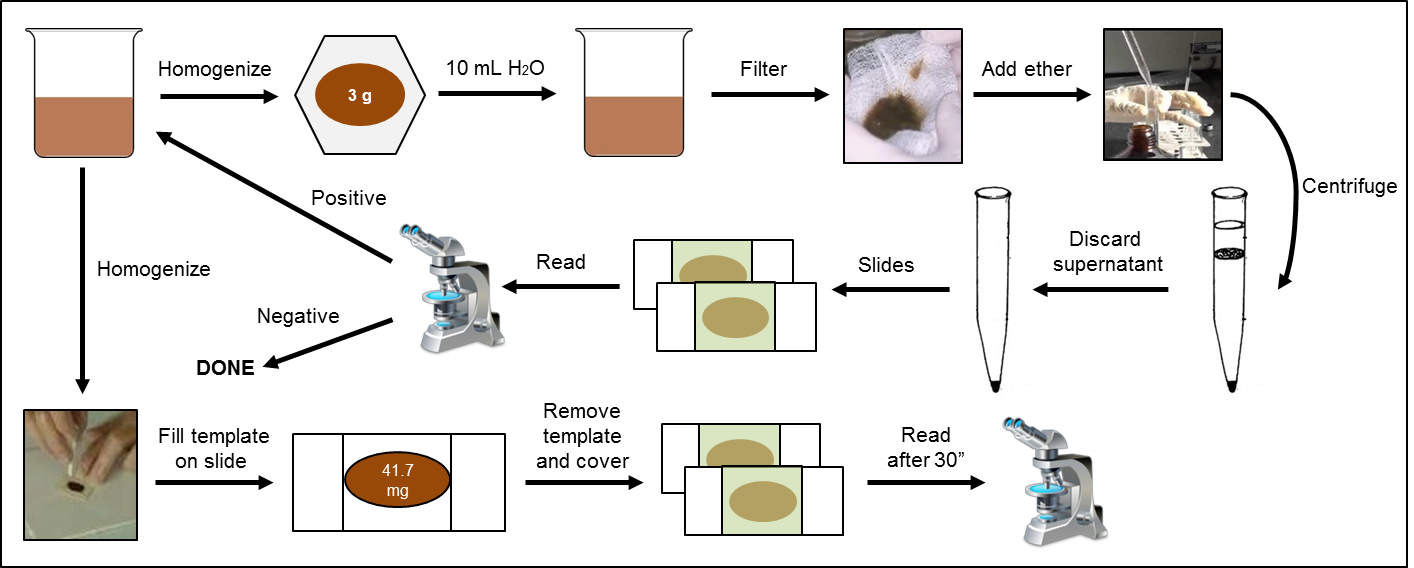

Supplement: S1 Fig — This standard could be used regardless of co-endemnicity. (TIF) [file pntd.0003967.s001.tif]
