# Supplementary material for: The Right Tool for the Job: Detection of Soil-Transmitted Helminths in Areas Co-endemic for Other Helminths
Source: PLoS Negl Trop Dis. 2015 Aug 4;9(8):e0003967. doi: 10.1371/journal.pntd.0003967 (PMC4524677; doi:10.1371/journal.pntd.0003967)
Supplement: S1 Flowchart — (DOC) [file pntd.0003967.s003.doc]

ENROLLMENT

n = 1331

ALLOCATION

n = 1260

ANALYSIS

n = 1260

FOLLOW-UP

n = 302

POSITIVES FOR STHs

n = 311

ANALYSIS

n = 302

Not meeting inclusion criteria

n = 71

Negatives for STHs

n = 949

TREATED

n = 311

No sample at follow-up

n = 7
